# Factors associated with carditis adverse events following SARS-COV-2-19 vaccination

**DOI:** 10.1017/S0950268825000329

**Published:** 2025-03-19

**Authors:** Kyung Hyun Min, Jun Hyeob Kim, Jin Yeon Gil, Jun Hyuk Park, Ji Min Han, Kyung Eun Lee

**Affiliations:** College of Pharmacy, Chungbuk National University, Cheongju, Republic of Korea

**Keywords:** COVID-19, machine learning, myocarditis, population-based study, vaccine safety

## Abstract

The study aimed to delve into the incidence and risk factors associated with myocarditis and pericarditis following SARS-COV-2-19 vaccination, addressing a notable gap in understanding the safety profile of vaccinations. Through meticulous data selection from the National Health Insurance System (NHIS) database of Korea, the researchers employed both a case-crossover study and a nested case-control design to analyze temporal patterns and risk factors related to carditis occurrences post-immunization. Key findings revealed a significant association between SARS-COV-2-19 vaccination and the occurrence of carditis, with a strong temporal correlation observed within 10 days post-vaccination. Noteworthy factors contributing to carditis risk included the duration between vaccination and carditis, specific comorbidities and medication use. The study concluded by recommending an extended post-vaccination surveillance duration of at least 10 days and underscored the importance of considering individual medical histories and concurrent medication use in assessing vaccine-induced carditis risk. This study might contribute to understanding vaccine safety profiles and emphasizes the significance of comprehensive post-vaccination monitoring protocols.

## Introduction

As SARS-COV-2-19 vaccinations have consistently shown their effectiveness and safety, there has been a notable decline in the incidence rate of SARS-COV-2-19 infections [1]. The increased vaccination rate has, however, been associated with an increase in the frequency of various side effects [2]. Among a range of adverse events (swelling, fever, fatigue, fever, headache, etc.), myocarditis and pericarditis, inflammatory processes affecting the cardiac muscle and the pericardial tissue respectively, have emerged as noteworthy concerns [2–4]. While often manifesting as mild or even favourably resolving clinical presentations, these conditions may, albeit infrequently, culminate in severe manifestations such as fulminant myocarditis and fatal outcomes [5–9]. Various factors, including but not limited to younger age, male gender, and occurrence after the administration of the second vaccine dose, have been proposed as potential determinants of carditis [8–11].

Despite the potential severity of such complications, there is a lack of thorough examination of a broad range of concomitant diseases, concurrent medication use, and the best period for post-vaccination surveillance. To fill this gap, the current study meticulously examined a large cohort of patients who were diagnosed with carditis after receiving the SARS-COV-2-19 immunization. In order to examine the temporal patterns underlying the incidence of carditis after immunization, as well as potential risk factors such as comorbidities and comedications, we utilized the robust National Health Insurance System (NHIS) database of Korea

## Materials and methods

### Data selection

We accessed the NHIS customized health information repository using information obtained from the National Health Insurance Sharing Service (NHISS) and the Korean Centers for Disease Control and Prevention. We systematically identified patients with confirmed diagnoses of COVID-19 between 8th October 2020, and 31st December 2021. In addition, we selected a COVID-19-free control group that was five times as large as the aforementioned patients, assuring age and gender congruence. The extensive records maintained within the NHIS database were then used to create a database for each subject that included data from the years 2008 through 2021. The database included diagnostic codes, medical procedures, prescriptions for medications, and demographic information for all inpatient and outpatient interactions falling under the purview of the NHIS. Subjects with missing data were not included in the study. This enormous data aggregation was made possible by Korea’s single-payer national health system, which offers a distinct advantage. In particular, prescriptions and treatments are coded using the national procedure-coding system and the Anatomical Therapeutic Chemical (ATC) classification, while diagnostic coding follows the International Classification of Diseases, 10th Revision (ICD-10). The study population in this research predominantly consists of Korean individuals. South Korea is largely a homogeneous society, and the vast majority of those covered by the national health insurance system, from which our data is derived, are Koreans.

### Study designs

Our investigative framework comprised two separate study designs that used the same dataset and were each intended to provide insight into a different aspect of our research question. The case-crossover study was used to establish a relationship between the occurrence of carditis and the SARS-COV-2-19 immunization. Then, the nested case-control design was used as the main design to identify factors associated with carditis in vaccinated patients.

#### Case-crossover study

The case-crossover design was used to confirm the relationship between SARS-COV-2-19 vaccination and carditis. Patients were included in the case group if they had an initial diagnosis of myocarditis or pericarditis after their first documented immunization date. The temporal dimensions of our analysis encompassed a 30-day hazard period, a 30-day wash-out period, and a 30-day control period, spanning over a 90-days prior to the first carditis diagnosis. Thus, we included patients with the first carditis diagnosis occurring at least 90 days after the vaccination. We employed a variety of features, including immunization type and order, comorbidity, and drug classification according to the ICD-10 code and Korea Pharmaceutical Information Center (KPIC) pharmaceutical classification. The case-crossover design, which is a self-controlled design, precluded consideration of sex and age.

#### Nested case-control design

The case group consisted of vaccinated patients who developed carditis within 30 days of receiving the vaccination. The index date was defined as the date of the first carditis diagnosis, and the duration was computed between the most recent vaccination date and the index date. Individuals without a carditis diagnosis lacked a corresponding index date. The index date for these patients was randomly assigned within the temporal bounds of vaccine records, up until 31st December 2021. Concurrently, patients with index dates within 30 days post-vaccination were selected as a pool for the control group. We conducted one-on-one matching by age and sex to select the final control group. The year prior to the vaccination date was examined for features. To assess every potential effect of each comorbidity and medication, we created features for each ICD-10 code and KPIC pharmaceutical classification. We performed sensitivity analyses using half-year and two-year examination periods to confirm the potential bias caused by using a one-year feature examining period.

A machine learning method was used to efficiently extract the important features from the large set of 2 321 features created. In preparation of the data, categorical features, such as vaccine type, vaccine order, and sex, were encoded using one-hot encoding, and continuous features were standardized using min-max scaling. Since categorical features were predominant, the CATBOOST gradient boosting model, which is designed for categorical data learning and prediction, was appropriate and was selected [12]. Through 5-fold cross-validation, the bias related to data division was minimized. Using randomized search, the most effective hyperparameters were discovered, leading to the model with the highest Area Under the curve of the Receiver Operating Characteristic (AUROC) score. We evaluated feature importances and then determined the top 30 features that were most effective in predicting the occurrence of vaccine-induced carditis. All machine learning procedures were performed using Python 3.11.4.

### Statistical analysis

The chi-square test was used to evaluate categorical features and the t-test was used to evaluate continuous features. The multicollinearity of categorical features was examined using Cramer’s V score, and the collinear boundary was determined to be at 0.4, which is regarded as being relatively strong [13, 14]. One of the feature pairs with significant collinearity was eliminated for better explicability. Multi-feature logistic regression with stepwise selection was used to calculate adjusted odds ratios (aORs) for the most important features. All statistical calculations were conducted using SAS Enterprise Guide 8.3.

### Ethics/ethical approval

The Chungbuk National University Institutional Review Board granted the required ethical approval for the study procedure (CBNU-202205-HRHR-0098).

## Results

### Patient selection

#### Case-crossover design

**Figure 1.**
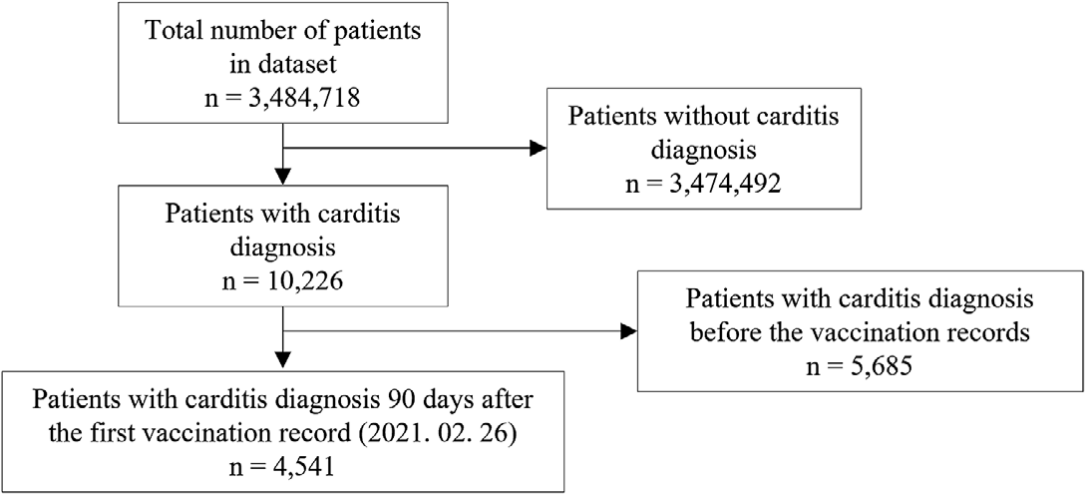
Flowchart of the patient selection in the case-crossover design.

A total of 10 226 cases of carditis were identified in the large cohort of 3 484 718 individuals. Among them, 4 541 patients received their initial carditis diagnosis at least 90 days after the immunization. The patient records from the hazard period and the control period each comprised the case group and the control group, respectively, due to the inherent self-control nature of the case-crossover design. A detailed flowchart is depicted in Figure 1.

#### Nested case-control design

**Figure 2.**
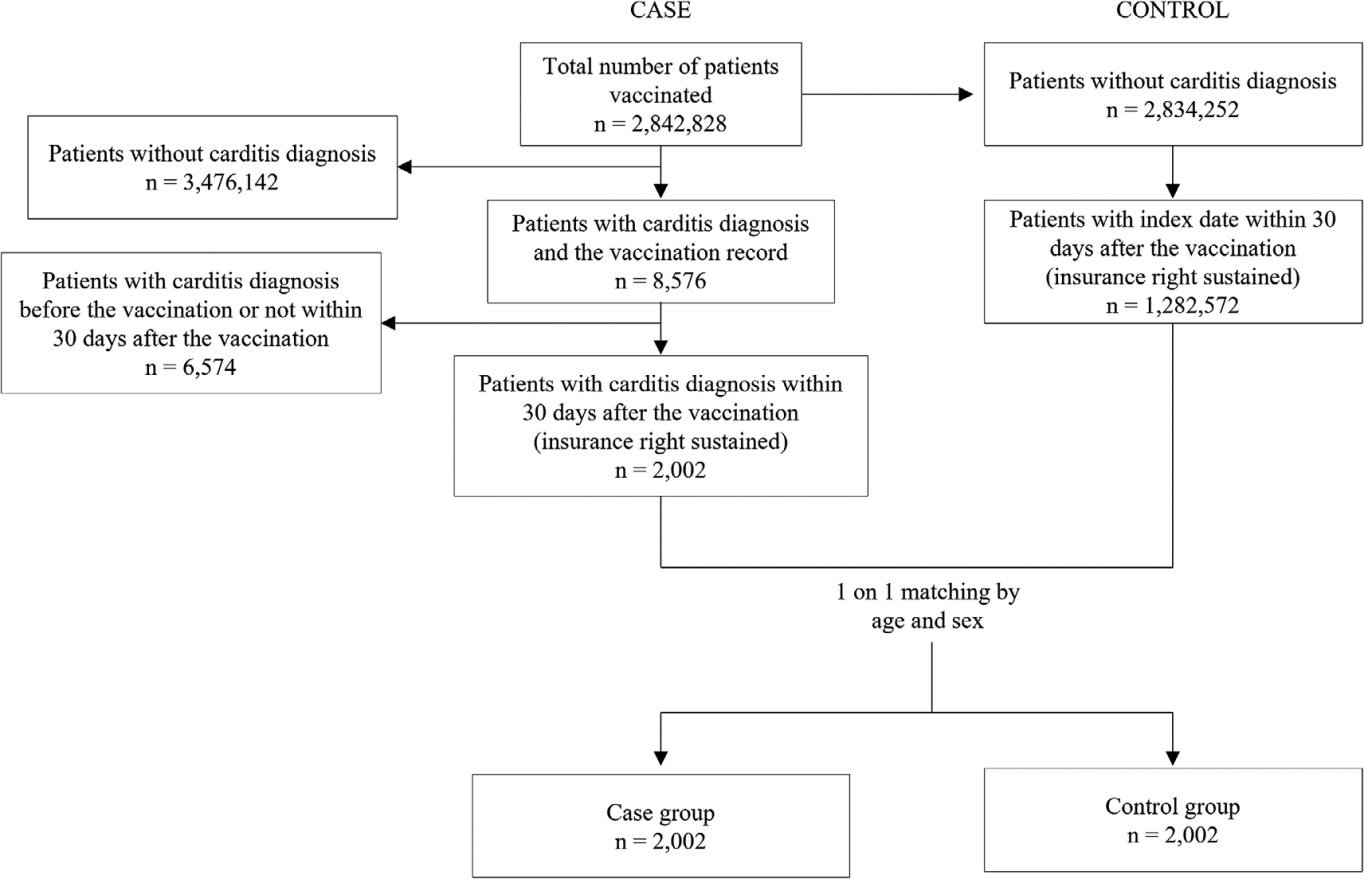
Flowchart of the patient selection in the nested case-control design.

Among patients who had received one or more vaccinations (n = 2 842 828), 8 576 patients with carditis were identified. Among them, the case group included 2002 patients who received a carditis diagnosis within 30 days following immunization. From the pool of 2 834 252 patients without carditis diagnoses, a random selection was made to designate index dates. Patients having index dates within 30 days post-vaccination were identified as the control pool (1 282 572 individuals). The 2002 matched controls were selected through one-on-one matching by age and sex. A detailed flowchart of nested case-control design is depicted in Figure 2.

### Patient characteristics in association with carditis

#### The case-crossover design

**Figure 3.**
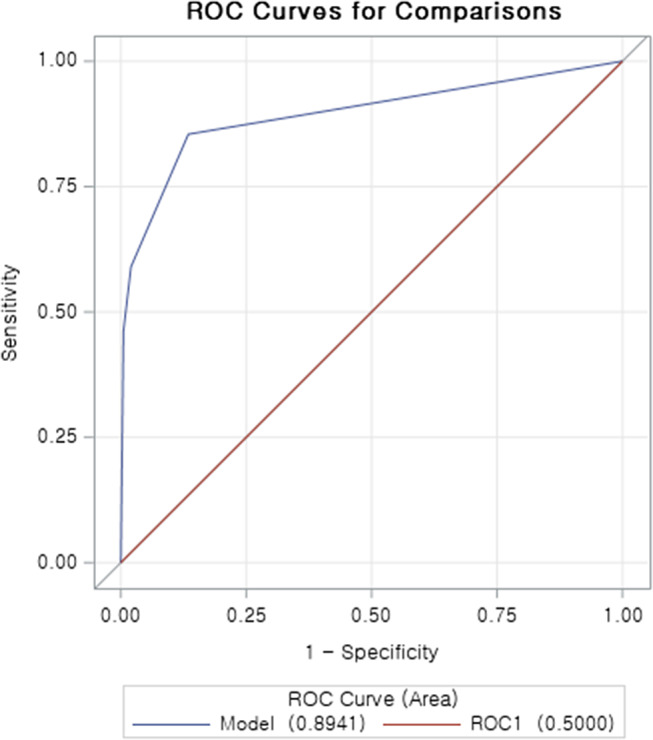
AUROC curve of the logistic regression model in the case-crossover design.

During the hazard period, 3 298 of the 4 541 patients showed a history of vaccination, as opposed to 543 patients during the control period. We calculated aORs of the features that showed the relationship between immunization and the development of carditis using strict adjustments that accounted for 2 321 comorbidities and comedications. Utilizing the logistic regression with stepwise selection, two features were retained: vaccination (aOR: 13.18, 95% confidence interval (CI): 11.56–15.02) and the disease code signifying pain in chest and throat (aOR: 47.85, 95% CI: 38.05–60.16). The AUROC score calculated for the logistic model was 0.8941 (p-value <0.0001, Figure 3).

#### The nested case-control design

**Figure 4.**
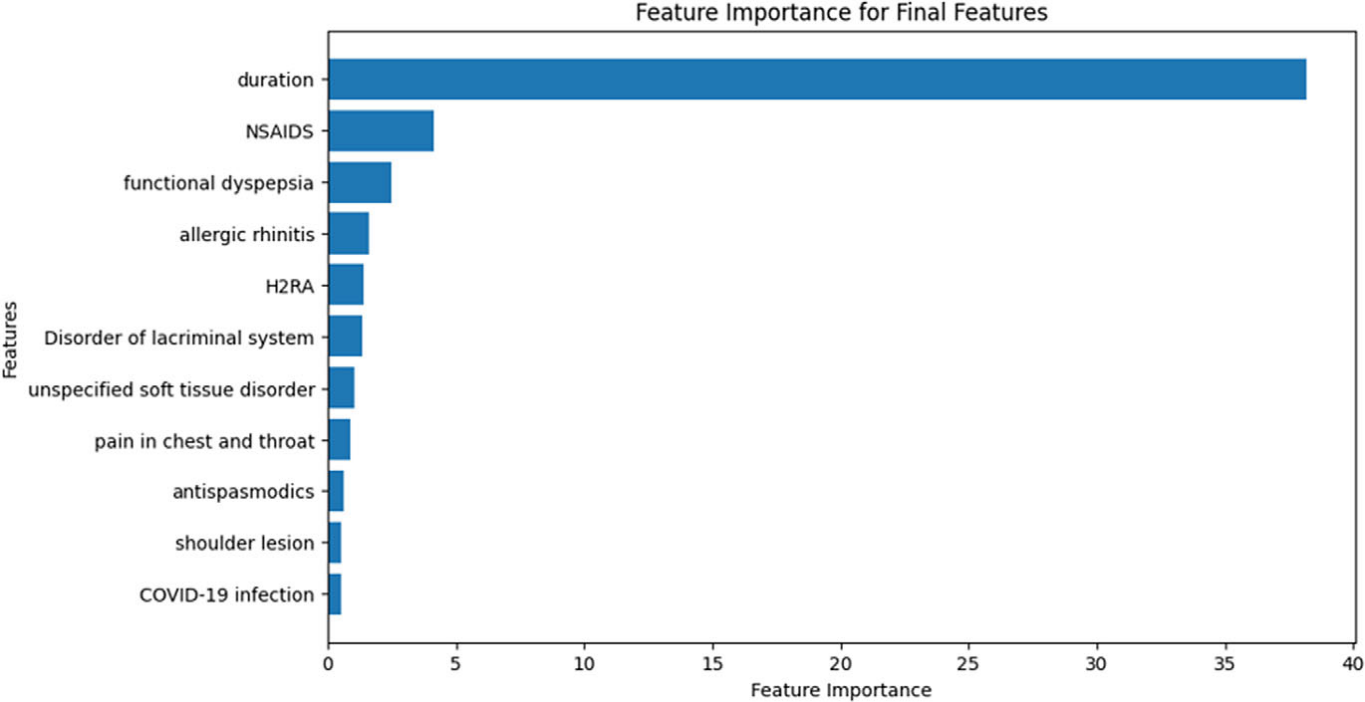
Calculated feature importances of the selected features by the machine-learning procedures.

**Figure 5.**
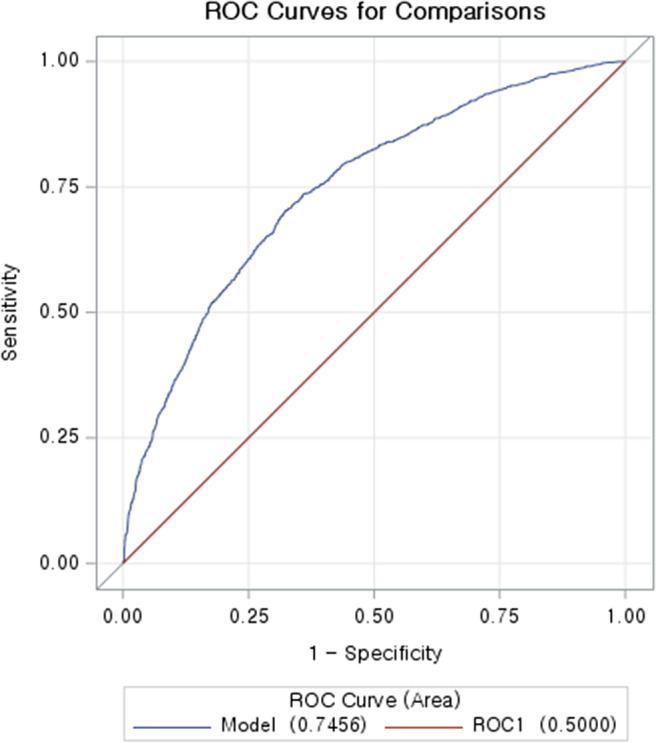
AUROC curve of the logistic regression model in the nested case-control design.

Based on the feature importance determined by machine learning procedures, 30 out of 2 321 features were selected. After eliminating one of the feature pairs with high collinearity, we conducted the multi-feature logistic regression with stepwise selection to select the final set of features. We included the baseline characteristics of selected features of the aforementioned procedure (Table 1). Other than the features used for matching and not included in the top 30 features selected by importance (age, sex, and vaccine information), all other features showed p-values below 0.0001 in chi-square and t-tests. The duration between vaccination and carditis emerged as the most important factor of the carditis event (Figure 4). The aOR for the duration was estimated at 0.952 (95% CI: 0.945–0.959, p-value: 0.0001), signifying a decrease in carditis risk with a longer interval following immunization. Carditis risk was uniformly increased by additional comorbidities and medication use. Functional dyspepsia, allergic rhinitis, disorders of the lacrimal system, unspecified soft tissue disorders, pain in the throat and chest, shoulder lesions, and SARS-COV-2-19 infection were associated with carditis incidents. Among comedications used, profens, H2 receptor antagonists, and antispasmodics were associated with carditis risk. Table 2 offers a description of aORs for the remaining features. The AUROC score calculated for the logistic model was 0.7456 (p < 0.0001, Figure 5).

**Table 1.** Baseline characteristics

Features	Case(n = 2002)	Control(n = 2002)	P-value
Sex (Male)	1 071 (53.50%)	1 071 (53.50%)	1.0000
Age, years	35.95 (±13.81)	35.95 (±13.81)	1.0000
10–19	174 (8.69%)	174 (8.69%)	1.0000
20–29	612 (30.57%)	612 (30.57%)	1.0000
30–39	510 (25.47%)	510 (25.47%)	1.0000
40–49	377 (18.83%)	377 (18.83%)	1.0000
50–59	199 (9.94%)	199 (9.94%)	1.0000
60–69	87 (4.35%)	87 (4.35%)	1.0000
70–79	32 (1.60%)	32 (1.60%)	1.0000
80–89	10 (0.50%)	10 (0.50%)	1.0000
≥90	1 (0.05%)	1 (0.05%)	1.0000
Vaccine type			<0.0001
Astrazeneca	47 (2.35%)	186 (9.29%)	<0.0001
Pfizer-BioNTech	1,513 (75.57%)	1 381 (68.98%)	<0.0001
Moderna	437 (21.83%)	402 (20.08%)	0.1741
Janssen	5 (0.25%)	33 (1.65%)	<0.0001
Vaccine order			0.0003
1st dose	1 047 (52.30%)	922 (46.05%)	<0.0001
2nd dose	819 (40.91%)	914 (45.65%)	0.0024
3rd dose	136 (6.79%)	166 (8.29%)	0.0726
Duration between vaccination and index date, days	9.87 (±7.85)	13.46 (±9.21)	<0.0001
Comorbidity			
Functional dyspepsia	713 (35.61%)	429 (21.43%)	<0.0001
Allergic rhinitis	873 (43.61%)	538 (26.87%)	<0.0001
Disorders of the lacrimal system	479 (23.93%)	287 (14.34%)	<0.0001
Unspecified soft tissue disorders	540 (26.97%)	308 (15.38%)	<0.0001
Pain in throat and chest	211 (10.54%)	95 (4.75%)	<0.0001
Shoulder lesions	179 (8.94%)	90 (4.50%)	<0.0001
SARS-COV–2–19 infection	202 (10.09%)	128 (6.39%)	<0.0001
Co-medication			
Profens	1,088 (54.35%)	705 (35.21%)	<0.0001
H2 receptor antagonist	743 (37.11%)	465 (23.23%)	<0.0001
Antispasmodics	471 (23.53%)	237 (11.84%)	<0.0001

**Table 2.** Adjusted odds ratios of final features after multivariable logistic regression with stepwise selection

Features	Adjusted odds ratios	95% Wald confidence limits		p-value
Duration between vaccination and the onset of the carditis event, days	0.952	0.945	0.959	<0.0001
Profens	1.408	1.211	1.637	<0.0001
Functional dyspepsia	1.335	1.140	1.564	0.0003
Allergic rhinitis	1.417	1.217	1.649	<0.0001
H2 receptor antagonist	1.266	1.082	1.483	0.0033
Disorders of the lacrimal system	1.504	1.265	1.788	<0.0001
Unspecified soft tissue disorders	1.265	1.061	1.508	0.0087
Pain in throat and chest	1.656	1.266	2.167	0.0002
Shoulder lesions	1.604	1.212	2.122	0.0009
SARS-COV–2–19 infection	1.327	1.036	1.700	0.0249
Antispasmodics	1.534	1.268	1.855	<0.0001

Adjusted for profens, GI motility regulator, H2 Receptor Antagonist, acetaminophen, corticosteroids, second-generation antihistamines, anticholinergics, antispasmodics, papaverines, adsorbent antidiarrheals, functional dyspepsia, disorders of refraction and accommodation, allergic rhinitis, disorders of lacrimal system, unspecified soft tissue disorders, pain in throat and chest, gastro-oesophageal reflux disease, keratitis, dizziness, shoulder lesions, SARS-COV-2-19 infection, acute bronchitis, retinal disorders, headache, other disorders of fluid, electrolyte, and acid–base balance.

**Figure 6.**
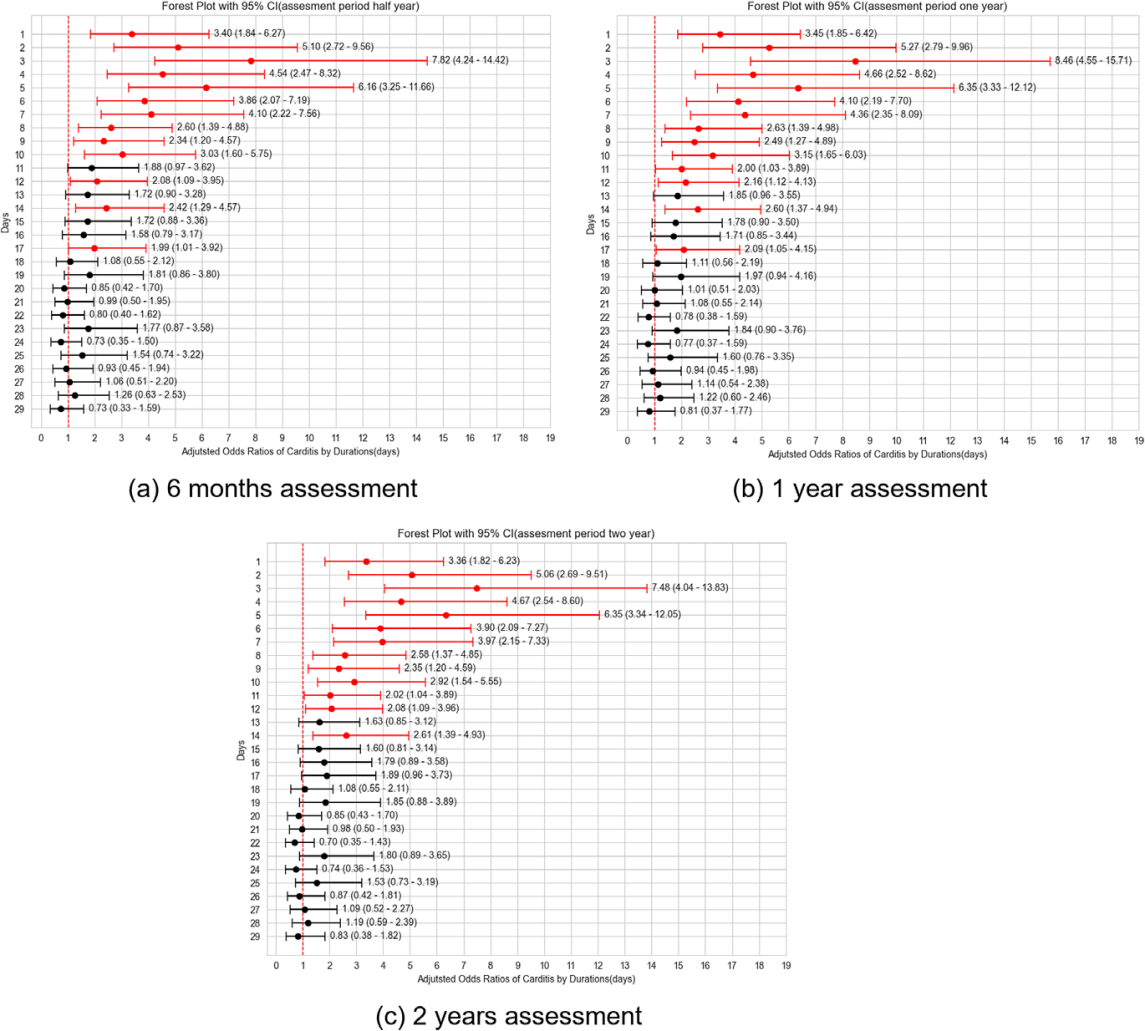
Comparison of post-vaccination daily carditis risk across sensitivity test. (a) 6 months assessment. (b) 1 year assessment. (c) 2 years assessment.

The temporal dynamics of carditis incidence after vaccination were thoroughly investigated. The probability of carditis incidence increased on day 1 after vaccination, peaked around day 3, and then gradually declined. Notably, aORs for the risk of carditis remained elevated above unity until Day 10, after which they entered the domain of statistical equivalence (95% confidence interval inclusive of 1.00). The observed trends persisted after conducting robust sensitivity analyses using multiple feature examination periods (six months and two years), supporting the stability of these findings. Calculated aOR in each sensitivity analysis is shown in Figure 6.

## Discussion

Since the SARS-COV-2-19 vaccine was introduced a few years ago, several studies have been published investigating the association between vaccine administration and vaccine-induced myo/pericarditis [10, 15–18]. We were able to identify observational studies that utilized database built by Asian countries [19–21]. In previous studies examining vaccine-induced carditis, factors such as age, sex, and the second vaccination were associated with carditis development [5, 10, 16, 17, 21, 22]. These studies focused primarily on examining the correlation between vaccine administration and carditis and were not specifically designed to identify risk factors of carditis in vaccinated patients. Our study sought to discover the factors associated with vaccine-induced carditis. To minimize bias and leverage the benefits of machine learning, we conducted an exploratory study that included a wide range of clinical conditions and medications (2 321 features), in contrast to other observational studies which included a limited set of characteristics. Age and sex are well-established risk factors for carditis related to the SARS-COV-2-19 vaccination. In our study, we controlled for these variables through matching techniques to focus on exploring less-known risk factors. This approach allows us to delve deeper into potential risk factors that may have been overshadowed by more prominent demographic variables. In the context of SARS-COV-2-19 vaccination in South Korea, it is important to note that recommendations are generally consistent across age groups. According to the Korea Disease Control and Prevention Agency (KDCA), the standard vaccination regimen, which includes the first and second doses, is recommended for individuals aged 5 years and older. This approach differs from some other countries where recommendations may vary more significantly by age or other demographic factors. In our study, this relatively uniform vaccination policy across age groups in Korea helps to minimize potential confounding effects that might arise from age-specific vaccination recommendations.

According to a recent study from South Korea, vaccine-induced carditis was observed in 659 individuals out of a population of 44 322 068, resulting in an incidence rate of 14 868 cases per million people [19, 23]. In comparison, a study conducted in the United States reported 15 cases among 2 392 924 individuals, corresponding to an incidence rate of 62 684 cases per million [24]. Similarly, a study from Denmark identified 69 cases of vaccine-induced carditis in a population of 3 981 109, resulting in an incidence rate of 17 332 per million [25]. In the United Kingdom, 167 cases were reported among 17 999 580 individuals, leading to an incidence rate of 9.278 cases per million [10]. These findings show the variability in the incidence rates of vaccine-induced carditis across different countries. The incidence rate observed in South Korea is lower than those reported in the United States and Denmark but higher than that in the United Kingdom. Therefore, conducting studies on the population in Korea was considered valid.

Prior to investigating the risk factors, we validated the association between carditis and SARS-COV-2-19 vaccination in the NHIS database. It would be meaningless to attempt to identify risk factors within this dataset if there were no correlation between vaccine administration and carditis occurrence. To achieve this, we adopted the case-crossover design, suitable for examining short-term side effect correlations. In this sub-study, a strong correlation (aOR: 13.18, 95% CI: 11.56–15.02) between vaccine administration and carditis was confirmed. Based upon the confirmation of vaccine-induced carditis, we conducted a nested case-control study using vaccine-administered patients as the ‘nest’ and used machine learning to identify key features associated with the risk of vaccine-induced carditis. To exclude false causality due to prodromal symptoms, all features were assessed not during the carditis event, but before vaccination.

In this study, the most influential feature of myocarditis occurrence within SARS-COV-2-19 vaccinated patients was the duration between vaccination and the carditis event. Other studies generally reported the incidence of carditis within approximately 2–4 days after the vaccination [8, 26]. However, the reported incidence duration in previous studies represented a statistical aggregation from observations made among patients who had developed carditis. The advantage of this study is that we directly selected duration as a potential factor and found a correlation regarding this factor. The duration feature spanned 1–30 days, and the aOR for the entire duration indicated a gradual decrease in carditis risk by about 5% per day (aOR: 0.952, 95% CI: 0.945–0.959). To investigate further, we explored the risk across different time spans in comparison to the reference duration of 30 days, which exhibited the lowest level of risk. The risk increased up to three days after vaccination, followed by a gradual decrease; however, the risk remained significantly high until Day 10. This trend remained consistent across various evaluation periods, as indicated by sensitivity analyses. The results of our study indicate that the carditis risk remains elevated for a more extended period of time than previously reported, suggesting the importance of monitoring beyond the initial 10 days after vaccination to detect an onset of carditis. In human and animal models of viral and autoimmune myocarditis, inflammation can develop up to 10–14 days after initial infection or immune response initiation, with peak myocardial inflammation typically observed around day 10. In humans, the disease progresses through acute (1–7 days, viral entry and innate immune response), subacute (1–4 weeks, adaptive immune response), and chronic phases, with similar stages observed in mouse models [27–32]. These findings suggest that the current short monitoring period following SARS-CoV-2 vaccination may be insufficient. Our study therefore proposes extending the monitoring period to at least 10 days post-vaccination to more effectively capture potential cases of vaccine-associated myocarditis and improve patient outcomes.

The 10 features other than duration were associated with an increased risk of carditis. Functional dyspepsia is a term encompassing difficulties in digestion, despite the absence of organic issues. Several studies have suggested that gastro-oesophageal reflux disease due to functional dyspepsia could provoke inflammation in the myocardium [33, 34]. The possibility of carditis arising from dyspepsia caused by *Helicobacter Pylori* infection has also been raised [33, 34].

SARS-COV-2-19 infection in vaccinated individuals was also associated with an increased incidence of carditis. When the SARS-COV-2-19 virus infects the lungs, it can activate numerous immune pathways that may impact the heart. This activation leads to the mobilization of immune cells such as mast cells, macrophages, B cells, and others throughout the body, potentially causing carditis as these cells cause inflammation in the heart [35].

Allergic rhinitis, disorders of the lacrimal system, unspecified soft tissue disorders, and shoulder lesions were identified as additional disease risk factors for carditis. Inflammation is a common characteristic of these four conditions. While viral infections such as SARS-COV-2-19 are a predominant cause of carditis, bacterial and fungal infections, inflammatory reactions to toxic substances, and systemic inflammatory diseases may also contribute to its increased incidence [32]. There is a possibility that the pronounced intergroup differences in these four inflammatory conditions are partly due to the large number of patients with these conditions in our database.

The use of profens, H2 receptor antagonists, and antispasmodics is associated with inflammation. Since we adopted efforts to remove multicollinearity, the statistical significance of these factors suggests that they have shown an independent effect from the diseases. These results suggest that among patients with the mentioned clinical conditions, those who used these medications and had more severe symptoms were at a higher risk of developing carditis.

The physical conditions mentioned earlier, such as allergic rhinitis and functional dyspepsia, were known to induce mast cell activation [36–38]. This phenomenon was similarly observed in SARS-CoV-2 infection [39–41]. Notably, mast cell activation was discovered as a significant factor in the development of carditis [29, 42, 43]. The increased risk of carditis associated with medications used to treat these physical conditions further supports the hypothesis that mast cell activation plays a crucial role in the pathogenesis of carditis.

It is the strength of this study that it applied features of all possible diseases and groups of medications (a total of 2 321 features) and ultimately selected the 11 most influential features through machine learning procedures. Through this process, all possible risk factors were explored and identified that could not have been anticipated prior to this research. Moreover, leveraging the extensive database of the Korean NHIS, constructed for 2 842 828 vaccine recipients, provided an opportunity to conduct research on a scale that is typically not accessible in standard clinical trials. Additionally, conducting sensitivity analyses within the main study design, along with preceding validation of the database, contributed to establishing credible results through a stringent research design.

A limitation of this study is the absence of certain data. The NHIS database consists of health insurance claims data, providing extensive coverage of both the number of individuals and all recorded diseases and medications claimed. However, it lacks clinical metrics due to the absence of laboratory data. Furthermore, although some health screening information is available, it does not cover all patients, making it challenging to reflect on lifestyle habits such as alcohol consumption and smoking.

SARS-COV-2-19 vaccination-related myo/pericarditis has a low incidence rate but can result in severe complications. Unlike previous studies, this study directly focused on exploring associated factors in vaccinated patients. The duration between vaccination and the carditis was found to be the most important factor, associated with a significant increase in risk up to 10 days post-vaccination. Consequently, we suggest monitoring carditis occurrence for at least 10 days following vaccination. We also found that carditis risk may increase due to inflammatory diseases and medications used. The findings of this study contribute to the understanding of vaccine-induced carditis in clinical practice.

## Data Availability

The data supporting the findings of this study are available from the Korean National Health Insurance Sharing Service. However, restrictions apply to the availability of these data, which were used with permission for this study.
